# Effect of COVID-19 pandemic on diagnosis and treatment of thyroid cancer in Brazil

**DOI:** 10.3389/fendo.2022.995329

**Published:** 2022-10-05

**Authors:** Vitor Bock Silveira, Wallace Klein Schwengber, Guilherme Moreira Hetzel, André B. Zanella, Rafael Selbach Scheffel, Ana Luiza Maia, Jose Miguel Dora

**Affiliations:** Thyroid Unit, Endocrine Division, Hospital de Clínicas de Porto Alegre, School of Medicine, Universidade Federal do Rio Grande do Sul, Porto Alegre, Brazil

**Keywords:** thyroid carcinoma, COVID-19, fine-needle aspiration (FNA) biopsy, thyroidectomies, radioiodine (131I) treatment

## Abstract

**Introduction:**

The COVID-19 pandemic delayed the diagnosis, treatment, and follow-up visits of patients with thyroid cancer. However, the magnitude with which these restrictions affected the Brazilian health care is still unknown.

**Methods:**

Retrospective analysis of thyroid cancer-related procedures performed in the Brazilian public health system from 2019 to 2021. Data were retrieved from the Department of Informatics of the Unified Health System (DATASUS). The following procedures were evaluated: fine-needle aspiration biopsies (FNABs), oncologic thyroidectomies, and radioiodine (RAI) therapies for thyroid cancer. The year of 2019 served as baseline control.

**Results:**

Compared with 2019, FNABs, oncologic thyroidectomies, and RAI therapies performed in 2020 decreased by 29%, 17% and 28%, respectively. In 2021, compared with 2019, FNABs increased by 2%, and oncologic thyroidectomies and RAI therapies decreased by 5% and 25%, respectively. Most pronounced reductions were observed in the first months of the pandemic. In April 2020, FNABs decreased by 67%, oncologic thyroidectomies by 45%, and RAI therapies by 75%. In 2021, RAI therapies were the only procedure with a statistically significant decrease.

**Conclusion:**

The restrictions to public health care during the COVID-19 pandemic resulted in a significant reduction in diagnostic and treatment procedures for thyroid cancer in Brazil. The effects of these transitory gaps in thyroid cancer care, due to COVID-19, are still unclear.

## Introduction

In late March 2020, community transmission of COVID-19 was identified within Brazil, and actions were taken to reduce exposure (1). Healthcare providers temporarily postponed cancer screenings, in-person consultations were shifted to telemedicine, and surgeries and other in-office procedures were delayed. In addition to restrictions and sparing of resources to fight an unprecedented health crisis, oncologic patients were oriented to keep distance from hospitals due to a greater risk of SARS-CoV-2 infection complications and death (2).

Most thyroid cancers are considered low risk and have an excellent prognosis (3). Hence, during the COVID-19 pandemic, medical societies released statements suggesting that fine-needle aspiration biopsies (FNABs), thyroid cancer surgeries, and radioiodine (RAI) therapies could be safely postponed without changes to individual prognosis for most cases (4, 5). Restrictions for these services were observed worldwide (6, 7). In Italy, data from 28 surgical units showed that the number of oncologic thyroidectomies decreased by 27.1% during the first wave of the COVID-19 pandemic (from March 2020 to August 2020) (8).

The Brazilian health system, a mix of public-private services, has three parts: the public (SUS), the private, and the private health insurance subsectors. Most of the Brazilian health services are provided by the public subsector, in which services are financed by the government (9). Indeed, data from 2019 show that more than 70% of Brazilians (approximately 150 million people) rely exclusively on the public subsector (10).

Since the peak of COVID-19, cases and hospitalizations have decreased, and it is time to look back and evaluate the effect those restrictions had on the diagnosis and treatment of thyroid cancer. We performed a retrospective analysis of thyroid cancer-related procedures, pre- and post-pandemic. The procedures analyzed were: FNABs, oncologic thyroidectomies, and RAI therapies.

## Methods

A retrospective analysis of thyroid cancer-related procedures was performed in the Brazilian public health system from 2019 to 2021. Data were retrieved from the Department of Informatics of the Unified Health System (DATASUS). The FNABs, oncologic thyroidectomies, and RAI therapies for thyroid cancer were analyzed. Data were collected following standardized procedure codes used in DATASUS (**Supplemental Table 1**). April 2020 was considered as the onset of the COVID-19 pandemic in Brazilian healthcare, and 2019 as the baseline control year. All comparisons included the period of January to December of each year.

Statistical analysis was performed with Statistical Package for the Social Sciences (SPSS) version 18 (IBM Corp., Armonk, NY). Data for continuous variables were expressed as mean ± standard deviation of the mean. Welch’s ANOVA was used with Games-Howell *post-hoc* test. Results were considered statistically significant if P-value < 0.05.

## Results

### Fine-needle aspiration biopsies

**Figure 1:**
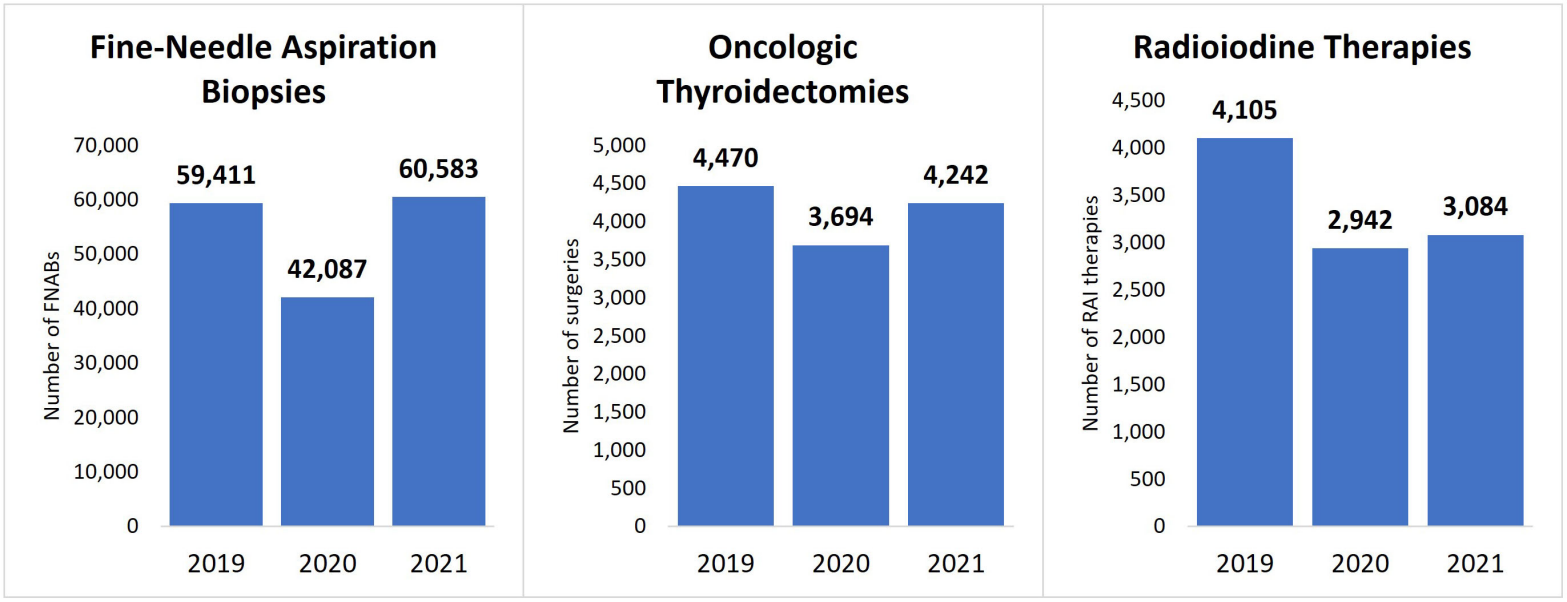
Number of procedures performed by year in Brazil’s public health system. FNABs, fine-needle aspiration biopsies; RAI, radioiodine therapy.

**Figure 2:**
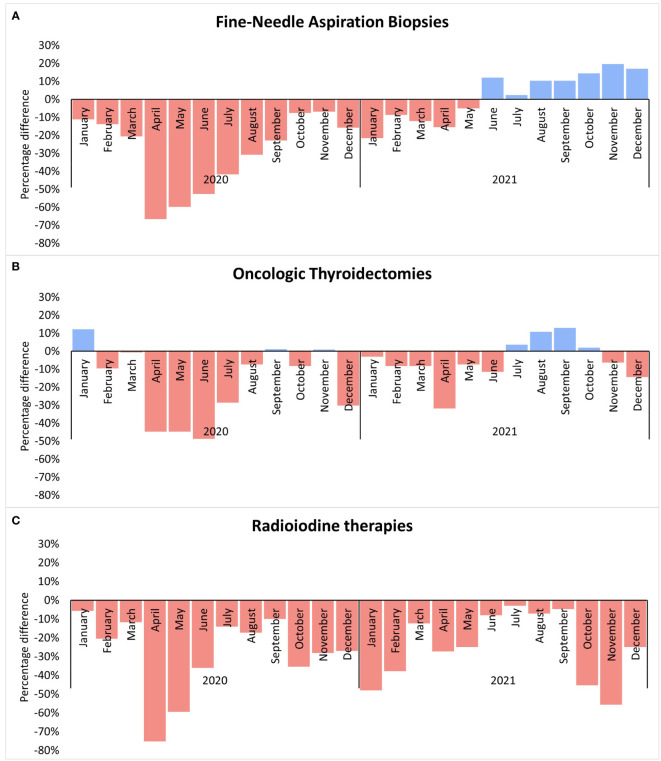
The percentage difference for each month of 2020 and 2021 in comparison with the 2019 monthly average of each procedure. **(A)** Fine-Needle Aspiration Biopsies; **(B)** Oncologic Thyroidetomies; **(C)** Radioiodine therapies.

**Figure 3:**
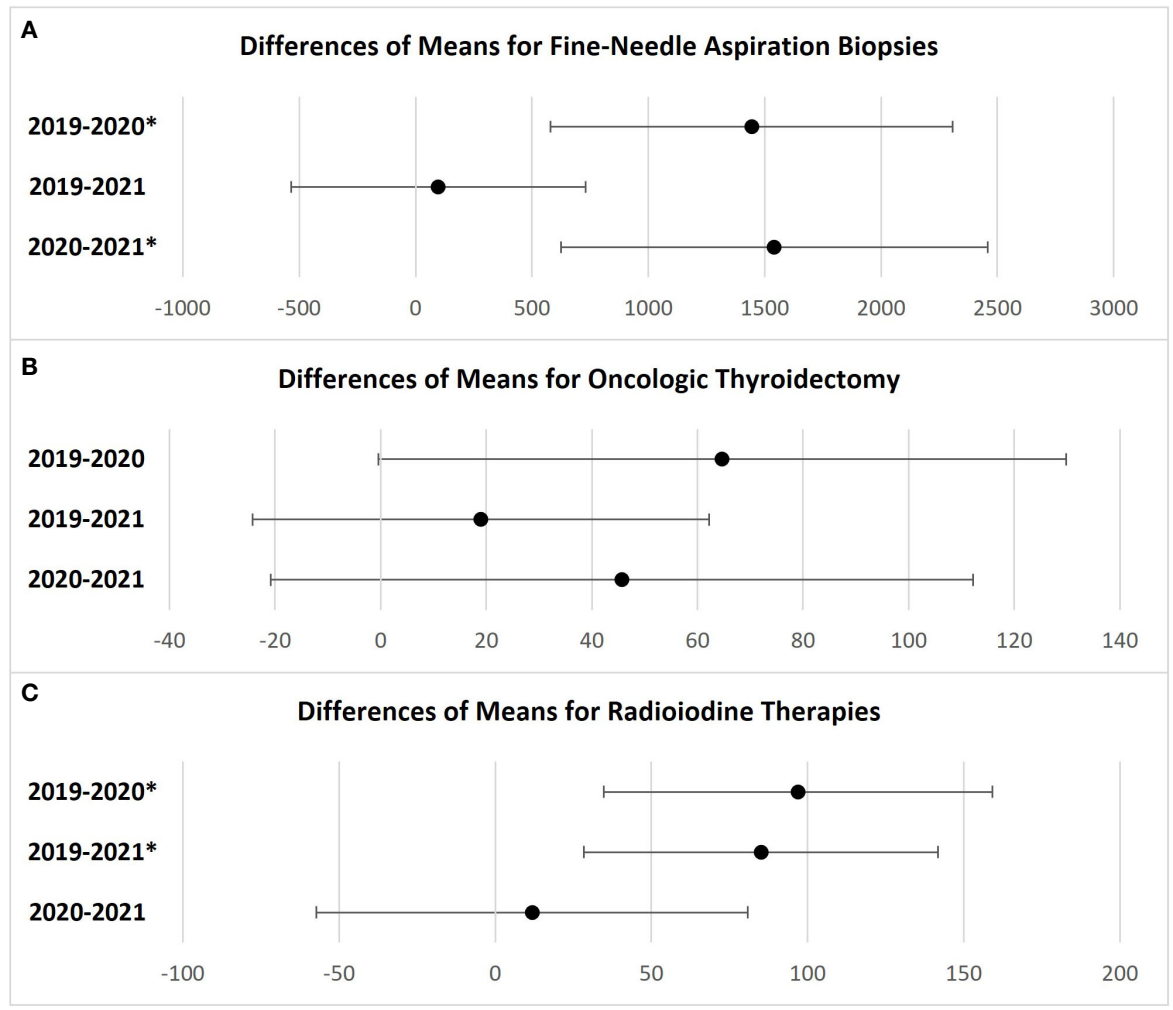
Games-Howell post-hoc test. Simultaneous 95% confidence intervals of differences between means of each procedure. **(A)** Fine-Needle Aspiration Biopsies; **(B)** Oncologic Thyroidetomies; **(C)** Radioiodine therapies. *Statistically significant. If an interval does not contain zero, the corresponding means are significantly different. Differences are expressed as absolute values.

In 2019, 59,411 FNABs were performed in the Brazilian public health system. The number of FNABs decreased to 42,087 (29% decrease) in 2020 and increased to 60,583 (2% increase) in 2021 (**Figure 1**). Comparing FNABs performed in 2020 and 2021 with the monthly average of FNABs performed in 2019, April 2020 represented the most pronounced decline (67% decrease). FNABs persisted below the monthly average of 2019 from April 2020 to May 2021 (**Figure 2A**). **Table 1** shows a summary of the data and statistical analysis. **Figure 3A** shows the *post-hoc* pairwise comparisons.

**Table 1:** Number of fine-needle aspiration biopsies, oncologic thyroidectomies, and radioiodine therapies in 2019, 2020, and 2021.

	2019	2020	2021
**FNABs (n)**	59,411	42,087	60,583
Mean FNABs per month*	4,951 ± 520	3,507 ± 1,040	5,049 ± 694
**Oncologic thyroidectomies (n)**	4,470	3,694	4,242
Mean thyroidectomies per month*	373 ± 39	308 ± 78	354 ± 45
**RAI (n)**	4,105	2,942	3,084
Mean RAI per month*	342 ± 46	245 ± 71	257 ± 63

Comparison between the monthly average of procedures of 2019, 2020, and 2021. Welch’s ANOVA test was used. *Statistically significant. FNABs: p<0.001; Oncologic Thyroidectomies: p = 0.025; RAI: p = 0.001.

FNABs, fine-needle aspiration biopsies; RAI, radioiodine therapy.

### Oncologic thyroidectomies

In 2019, 4,470 oncologic thyroidectomies were performed in the Brazilian public health system. The number of oncologic thyroidectomies decreased to 3,694 (17% decrease) in 2020 and to 4,242 (5% decrease) in 2021 (**Figure 1**). The greatest reductions occurred from April to July 2020, with new significant drops in December 2020 and April 2021 (**Figure 2B**). **Table 1** shows a summary of the data and statistical analysis. **Figure 3B** shows the *post-hoc* pairwise comparisons.

### Radioiodine therapy

In 2019, 4,105 RAI therapies were performed in the Brazilian public health system. The number of RAI therapies decreased to 2,942 (28% decrease) in 2020 and to 3,084 (25% decrease) in 2021 (**Figure 1**). Since January 2020, all months had fewer RAI therapies than the monthly average of 2019. April 2020 presented the greatest reduction (**Figure 2C**). **Table 1** shows a summary of the data and statistical analysis. **Figure 3C** shows the *post-hoc* pairwise comparisons.

## Discussion

Our study aimed to evaluate the effect of COVID-19 on thyroid cancer care in Brazil. The number of FNABs, oncologic thyroidectomies, and RAI therapies largely decreased over the first few months of the pandemic. Despite following an increasing trend thereafter, oncologic thyroidectomies and RAI procedures have not returned to pre-pandemic levels.

In the context of diagnosis, experts in the area recommended that FNABs be deferred for most asymptomatic thyroid nodules (5). In Brazil, 17,324 (−29%) fewer FNABs were performed in 2020 in comparison to 2019. Considering that 6% of all FNABs are classified as Bethesda V or VI (11), the number may imply that more than 1,000 malignant or suspicious for malignancy lesions were not diagnosed.

In 2021, the number of FNABs was similar to pre-pandemic levels, despite still having new reductions from January to May 2021, as seen in **Figure 2A**. However, a consistent increase in FNABs procedures from June to December 2021 is observed, and it compensated for the prior reductions. Possibly, FNABs had a faster recovery because they are not dependent on hospital beds.

On the other hand, we should consider that, to some extent, nodules with relevant clinical and ultrasound features must have been prioritized during periods of service restrictions. In Brazil, the number of oncologic thyroidectomies decreased by 17% and 5% in 2020 and 2021, respectively, when compared to 2019. These results did not achieve statistical significance when we compared the monthly average of oncologic thyroidectomies (**Figure 3B**). The smaller reductions observed in oncologic thyroidectomies, compared with FNABs and RAI therapies, could be attributed to efforts to prioritize patients in more need of care.

Brazilian data shows that almost one-third of RAI therapies for thyroid cancer were postponed in 2020. The greatest reductions were in the first two months of service restrictions: a 75% reduction in April and a 59% reduction in May 2020. Different from FNABs and oncologic thyroidectomies, the number of RAI therapies had a significant reduction in 2021 as well. There was a 25% drop for RAI therapy in 2021 when compared with 2019, a percentage close to what was observed in 2020. We hypothesize that this discrepancy is due to disruptions observed in the national RAI supply chain in 2021 (12). Moreover, previous studies showed that there is a great disparity in the availability of RAI therapies between Brazilian states (13). Therefore, it is possible that the COVID-19 outbreak, and supply chain disruptions decreased the availability of this resource in states which were already underserved.

Efforts to reestablish thyroid cancer care to pre-pandemic levels should not result in low-value care. Thyroid cancer overdiagnosis due to the widespread use of diagnostic procedures has also been present in developing countries, including Brazil (14). In the context of current thyroid cancer epidemiology being composed mostly of low-risk tumors, some authors suggest that the effect the COVID-19 pandemic had on thyroid cancer management may serve as an opportunity to implement more conservative treatment options on a large scale, such as active surveillance (15). Our study quantified numbers of thyroid cancer-related procedures showing that COVID-19 disrupted thyroid cancer management patterns in a high magnitude. Nevertheless, little is known about the consequences due to the unprecedented nature of this pandemic. Thus, in addition to strategies to manage a growing amount of patients, discouragement of thyroid exams/procedures related to low-value care is timely (16, 17).

Additionally, thyroid cancer patients have suffered from more pronounced emotional and psychological distress when compared to the general population since the COVID-19 outbreak, as demonstrated in surveys conducted in China (18) and Italy (19). In this matter, appropriate psychological support is also necessary.

This study has limitations. The DATASUS database is composed of aggregated ecological data. Thus, since DATASUS was not designed as a cancer registry, it is prone to bias and does not include information about patients’ clinical and oncological features. Notwithstanding, considering that the DATASUS system is based on billing information, it is audited by competent authorities, which contributes to a minimal curation of the data. Additionally, our study encompasses data retrieved from the public health system, with no records from the private subsector or the private health insurance subsector. The SUS, however, is the world’s largest public health system, providing health care coverage for a population of 210 million people. This study aimed to measure the effect of the COVID-19 pandemic on thyroid cancer procedures in a developing country at a national level.

This study shows that the restrictions during the COVID-19 pandemic resulted in a significant reduction in diagnostic and treatment procedures for thyroid cancer within Brazil’s public health system. The effects of these transitory gaps in thyroid cancer care due to COVID-19 are still unclear.

## Data availability statement

The original contributions presented in the study are included in the article/**Supplementary Material**. Further inquiries can be directed to the corresponding author.

## Author contributions

VS, WS, GH, RS, and JD contributed to the study conception and design, data analysis and interpretation, and manuscript preparation. AZ and AM contributed to data analysis and interpretation, and manuscript preparation. All authors contributed to the article and approved the submitted version.

## Funding

Our study is supported by the Rio Grande do Sul Research Support Foundation (FAPERGS), Research Incentive Fund of the Hospital de Clínicas de Porto Alegre (FIPE/HCPA), and Programa Institucional de Bolsas de Iniciação Científica da Universidade Federal do Rio Grande do Sul (PIBIC/UFRGS).

## Conflict of interest

The authors declare that the research was conducted in the absence of any commercial or financial relationships that could be construed as a potential conflict of interest.

## Publisher’s note

All claims expressed in this article are solely those of the authors and do not necessarily represent those of their affiliated organizations, or those of the publisher, the editors and the reviewers. Any product that may be evaluated in this article, or claim that may be made by its manufacturer, is not guaranteed or endorsed by the publisher.
