# Supplementary material for: Effect of COVID-19 pandemic on diagnosis and treatment of thyroid cancer in Brazil
Source: Front Endocrinol (Lausanne). 2022 Oct 5;13:995329. doi: 10.3389/fendo.2022.995329 (PMC9581141; doi:10.3389/fendo.2022.995329)
Supplement: Supplementary file 1 [file Table_1.pdf]

Supplemental Table 1.

Procedure	DATASUS Code(s)
**Fine-needle aspiration biopsy**	**0201010470**
**Total thyroidectomy in oncology**	**0416030270**
**Total thyroidectomy with lymph node resection in oncology**	**0416030122**
**Total thyroidectomy with lymph node resection**	**0402010051**
**Transsternal thyroid tumor resection in oncology**	**0416030360**
**Radioiodine therapy 30 mCi**	**0304090050**
**Radioiodine therapy 50 mCi**	**0304090069**
**Radioiodine therapy 100 mCi**	**0304090026**
**Radioiodine therapy 150 mCi**	**0304090018**
**Radioiodine therapy 200 mCi**	**0304090034**
**Radioiodine therapy 250 mCi**	**0304090042**

Data source: TABNET/DATASUS (tabnet.datasus.gov.br)

Period: 2019–2021
